# Bigger Than a Hen’s Egg: A Case of Bouveret Syndrome

**DOI:** 10.7759/cureus.58742

**Published:** 2024-04-22

**Authors:** Eli A Zaher, Mohamed A Ebrahim, Omar Al Salman, Parth Patel, Marwah Alchalabi

**Affiliations:** 1 Internal Medicine, Ascension Saint Joseph Hospital, Chicago, USA; 2 Gastroenterology, Ascension Saint Joseph Hospital, Joliet, USA

**Keywords:** cholecystoduodenal fistula, upper endoscopy, cholelithiasis, gallstone ileus, bouveret's syndrome

## Abstract

Bouveret syndrome, a rare complication of cholelithiasis resulting in gallstone ileus, presents diagnostic and therapeutic challenges due to its low incidence and nonspecific symptoms. We report a case of Bouveret syndrome in a middle-aged male without significant medical history, emphasizing the need for heightened clinical suspicion. Diagnostic imaging, including computed tomography and upper endoscopy, revealed gastric outlet obstruction and a cholecystoduodenal fistula. Treatment involved unsuccessful endoscopic lithotripsy followed by surgical intervention. This case underscores the importance of interdisciplinary collaboration for successful management. With no standardized approach, individualized treatment strategies, including endoscopic and surgical interventions, are crucial for favorable outcomes in Bouveret syndrome.

## Introduction

Bouveret syndrome is a rare complication of cholelithiasis resulting from an acquired fistula between the gallbladder and the stomach or duodenum resulting in gallstone ileus. It occurs in about 0.3-0.5% of patients with cholelithiasis, and only in 1-3% of those with gallstone ileus. Obstruction tends to occur most commonly in the distal small bowel, with the least frequent cases occurring in the stomach or duodenum [1]. Only a small number of cases have been described worldwide, leading to a lack of concrete guidelines for diagnosis and management. Its non-specific presentation and high incidence in the elderly with multiple comorbidities further complicates treatment and contributes to its high mortality rate [2]. We present a unique case of Bouveret syndrome occurring in a middle-aged male without a significant past medical history.

## Case presentation

A 50-year-old non-obese male with no significant past medical history presented to the emergency department with intractable vomiting and diarrhea beginning two days prior. He ate at a new restaurant on the day of symptom commencement with his girlfriend, who was asymptomatic. His vomiting and diarrhea were non-bloody and without specific exacerbating or relieving factors. No fevers or chills were reported. He denied alcohol use or having similar symptoms in the past. He likewise denied using any medications or supplements.

**Figure 1:**
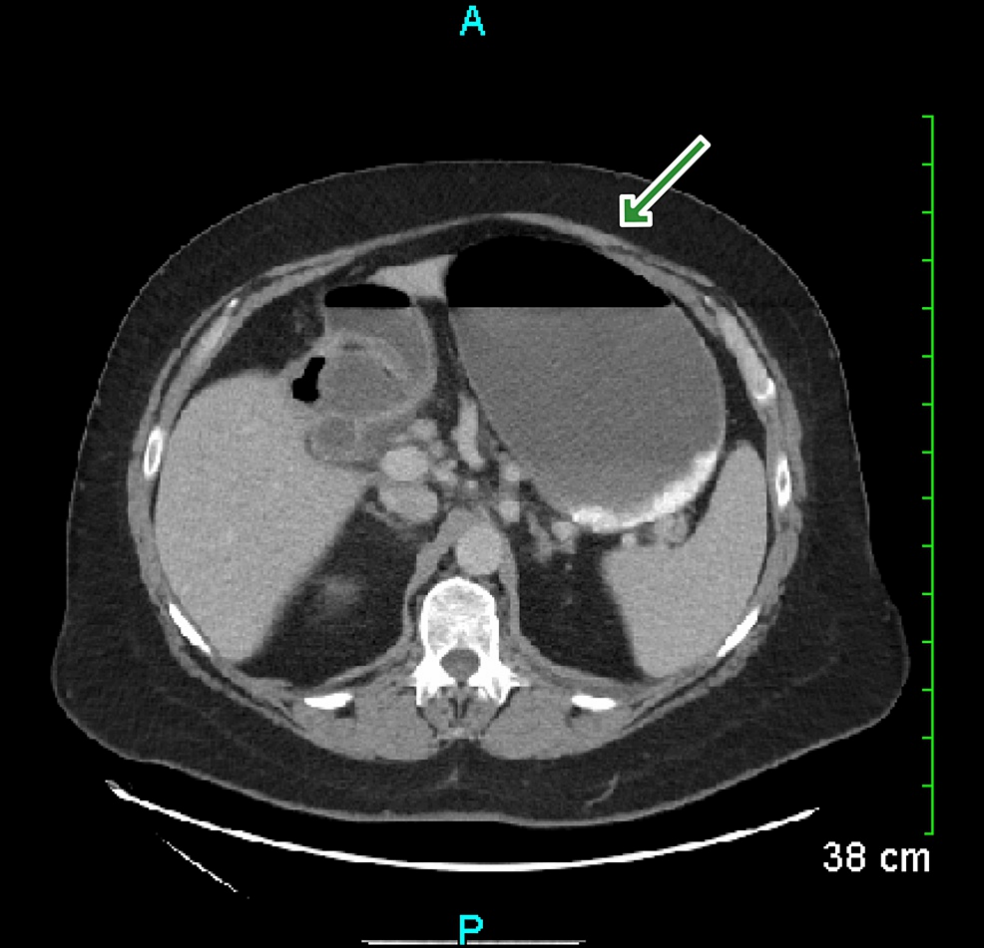
CT abdomen Green arrow pointing towards a severely distended stomach with air-fluid level.

On admission, his vitals were consistent with tachycardia of 110 bpm and an elevated blood pressure of 156/119 mm Hg. No fevers were measured. Blood work-up was remarkable for leukocytosis of 11.7 k/mm cu (reference range: 4.0-11.0 k/mm cu) and an alkaline phosphatase of 108 IU/L (reference range: 35-104 IU/L), but was otherwise normal. Computed tomography (CT) of the abdomen showed gastric outlet obstruction and severe dilation of the proximal stomach with air-fluid level (Figure 1).

**Figure 2:**
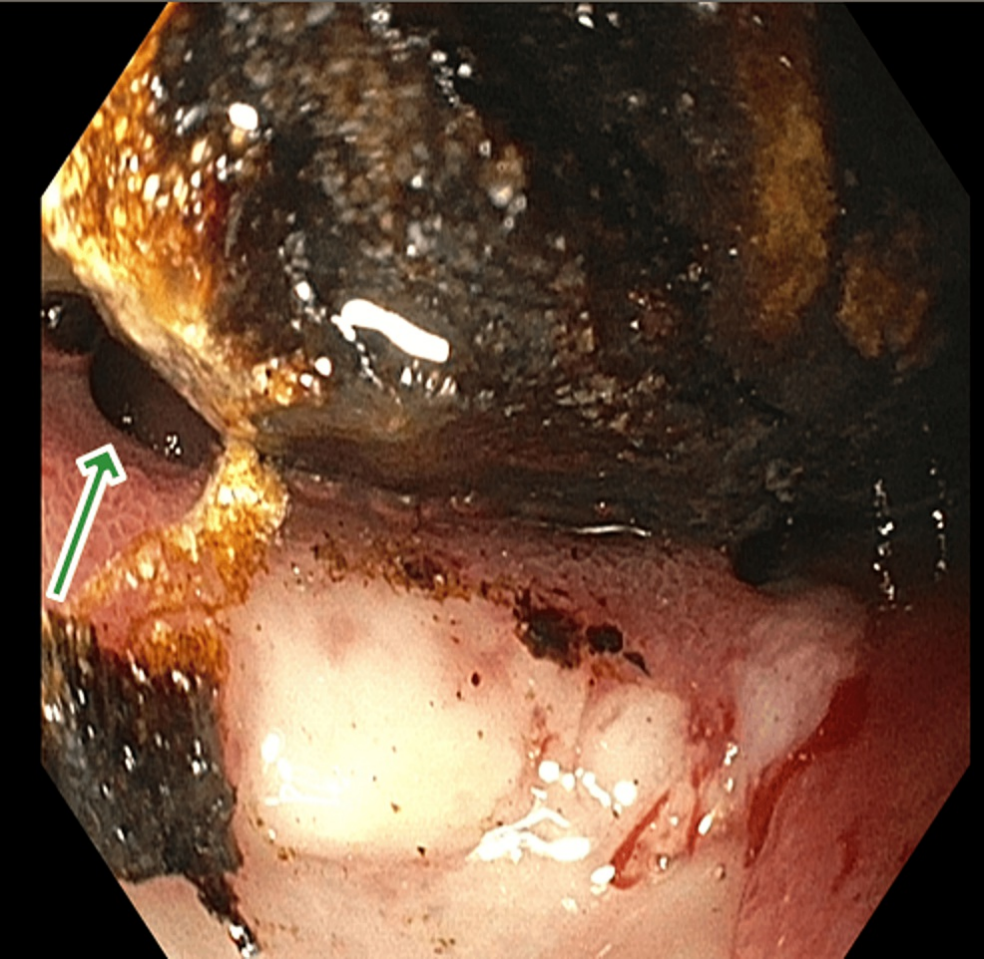
Upper endoscopy with a large gallstone in the duodenal bulb Green arrow pointing towards the cholecystoduodenal fistula with visible bile flow.

**Figure 3:**
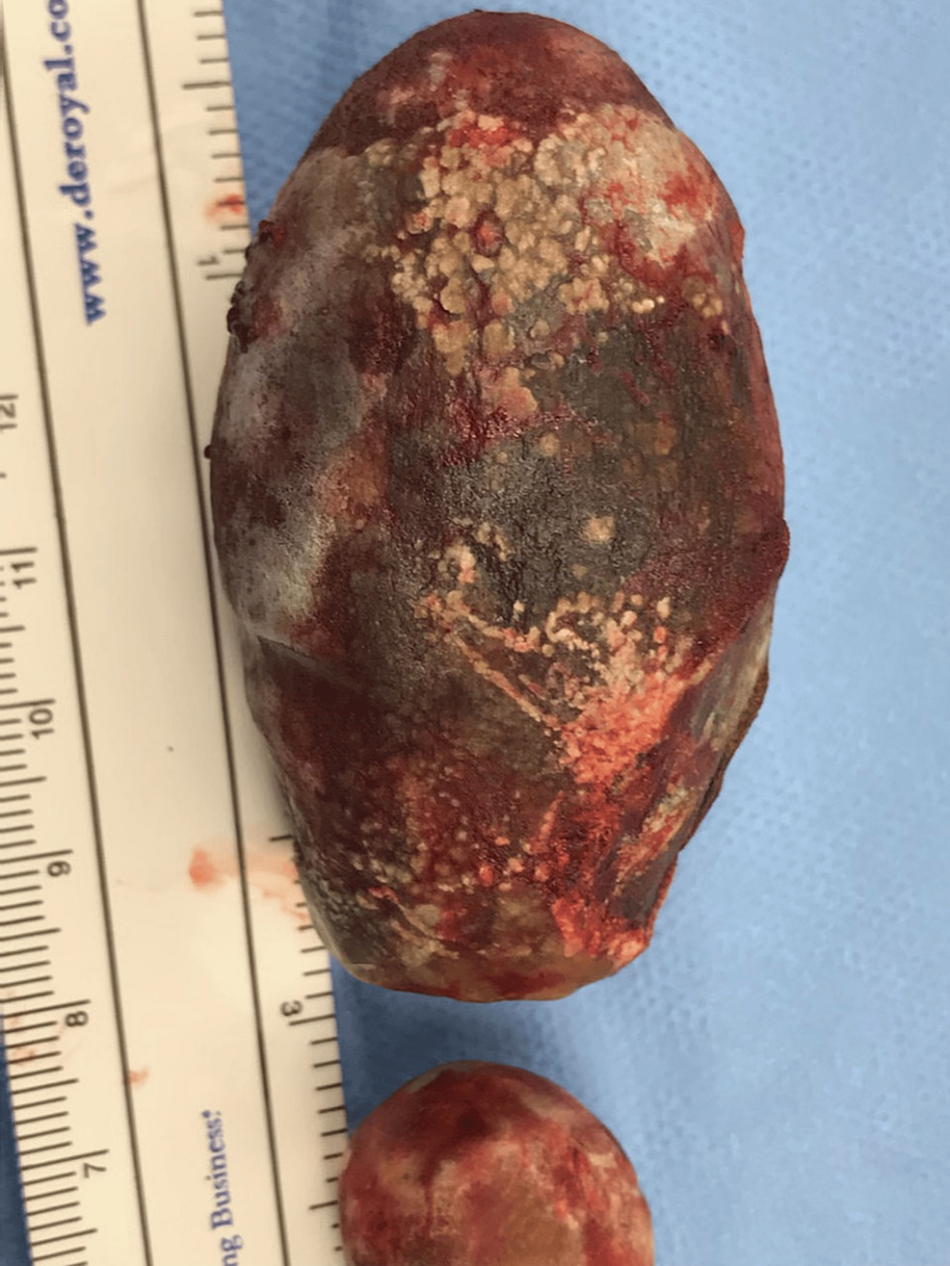
Post-operative picture of the gallstone

Based on the above findings, the patient was placed nil per os with a nasogastric tube for decompression. Gastroenterology was consulted and scheduled an upper endoscopy to rule out mechanical obstruction. Upper endoscopy revealed a large impacted stone within the duodenal bulb and a cholecystoduodenal fistula (Figures 2-3).

Following unsuccessful mechanical lithotripsy, general surgery was consulted and performed an exploratory laparotomy with repair of the fistula, removal of the stone, and a cholecystectomy.

## Discussion

The initial documentation of this rare condition dates back to Leon Bouveret, who published two case reports in Revue de Médecine in 1896 [2]. Diagnosis remains challenging due to the vague nature of symptoms and subtle physical examination findings. Recognizing Bouveret syndrome pivots on maintaining a high suspicion level in individuals with a history of gallstones and signs of gastric outlet obstruction. Typically, affected individuals are elderly women with various underlying health issues, including gallstone history, who present symptoms of bowel or gastric outlet obstruction. According to the most extensive analysis involving 128 cases, more than 85% of diagnosed patients experienced nausea and vomiting, with abdominal pain observed in around 70% of cases. Though less frequent, symptoms mimicking gastrointestinal bleeding, such as hematemesis (15%) and/or melena (6%), may also manifest [3]. Pathophysiologically, gallbladder inflammation and malignancy is believed to cause ischemic tears to the gallbladder and bowel wall, resulting in a fistula [2].

Most patients suspected of having a gastrointestinal tract obstruction typically undergo screening with an abdominal X-ray. Indications of Bouveret syndrome include Rigler's triad: a distended stomach, pneumobilia, and a visible opaque shadow indicating an enteric gallstone. For individuals experiencing pain in the right upper abdomen, ultrasound may be utilized. This examination may reveal the presence of gallstones with or without indications of cholecystitis, such as thickening of the gallbladder wall or fluid surrounding it. An upper gastrointestinal series, coupled with oral contrast, can provide further insight into an obstructive mass by revealing abnormalities like a filling defect, gallstone, stomach or duodenal dilation, pneumobilia, and/or outlet blockage [2,4]. In uncommon instances, there might be leakage of contrast material into the gallbladder, indicating the presence of a patent cholecystoduodenal or cholecystogastric fistula. Various imaging techniques, such as CT or MRI, are commonly employed to diagnose Bouveret syndrome, often revealing Rigler's triad and potential extravasation of contrast into the gallbladder, with fistula or pneumobilia present in most cases and gallstones seen in about half. Hematemesis prompts upper endoscopy, yet endoscopic diagnosis can be hindered by obscured visualization due to active bleeding or old clots, though obstruction evidence is usually evident. While multiple imaging methods are available, CT and EGD are favored due to their heightened sensitivity and specificity. Upon reviewing clinical cases, nearly all initial endoscopic examinations revealed signs of obstruction, yet visualization of the gallstone was only successful in about two-thirds of instances [3,4].

When stones cannot be easily extracted using an endoscopic net or basket, mechanical lithotripsy serves as a beneficial addition to crushing the stone before extraction. Typically, this method is employed to crush stones located in the stomach or upper part of the small intestine, followed by the removal of fragmented stone pieces through endoscopy [5]. Mechanical fragmentation can be achieved using tools such as a basket, snare, forceps, or a mechanical lithotripter. If malignancy is suspected, biopsies should be taken during endoscopy. It's crucial to ensure the removal of all stone fragments post mechanical fragmentation to prevent complications like gallstone ileus after surgery [6]. Laser lithotripsy, using techniques like neodymium: YAG, rhodamine, and holmium: YAG, is a promising method for breaking down stones with minimal tissue damage. It precisely targets the stone with focused energy, and some devices can differentiate between stone and tissue to prevent harm. Successful cases have shown the removal of large stones from the duodenum [7]. Initially, gallstones are extracted into the stomach with a basket, but if intact removal fails, a holmium: YAG laser is used through an endoscope's port to break them down. This method offers the advantage of applying high energy through small, flexible probes and has been used even after unsuccessful mechanical lithotripsy attempts [8]. If endoscopic treatment fails or isn't feasible due to a lack of expertise, surgical interventions may be necessary for managing patients with Bouveret syndrome, although it comes with a higher morbidity risk. These surgical options include open gastrotomy, pyloromyotomy, or duodenotomy at or near the point of obstruction [9-11]. Gastrotomy can be employed to extract the stone if it's feasible to maneuver an impacted gallstone from the duodenum into the stomach [12].

## Conclusions

Bouveret syndrome is a very rare condition affecting individuals with gallstones. Diagnosis often requires a high level of suspicion, especially in elderly women with gallstones, and can sometimes be discovered incidentally during imaging for symptoms of gastric outlet obstruction. Effective management involves close collaboration between therapeutic endoscopists and surgeons. While there's no standardized approach for treatment, initial diagnosis and potential therapy often start with endoscopy. In cases where gallstones are impacted and larger than 2-3 cm, multiple endoscopic techniques, including lithotripsy, may be necessary for successful removal. If endoscopy fails or expertise is lacking, surgical intervention is usually necessary.
